# A generalized right truncated bivariate Poisson regression model with applications to health data

**DOI:** 10.1371/journal.pone.0178153

**Published:** 2017-06-06

**Authors:** M. Ataharul Islam, Rafiqul I. Chowdhury

**Affiliations:** 1 Applied Statistics, East West University, Dhaka-1212, Dhaka, Bangladesh; 2 ISRT, University of Dhaka, Dhaka, Bangladesh; Fundacao Oswaldo Cruz, BRAZIL

## Abstract

A generalized right truncated bivariate Poisson regression model is proposed in this paper. Estimation and tests for goodness of fit and over or under dispersion are illustrated for both untruncated and right truncated bivariate Poisson regression models using marginal-conditional approach. Estimation and test procedures are illustrated for bivariate Poisson regression models with applications to Health and Retirement Study data on number of health conditions and the number of health care services utilized. The proposed test statistics are easy to compute and it is evident from the results that the models fit the data very well. A comparison between the right truncated and untruncated bivariate Poisson regression models using the test for nonnested models clearly shows that the truncated model performs significantly better than the untruncated model.

## Introduction

The bivariate Poisson models have emerged to address an increasingly important area of research with a wide range of applications in various fields where paired count data are correlated. Data on paired counts arise commonly in various areas of research such as traffic accidents (number of accidents and fatalities), medical research (number of visits to health professionals and utilization of health care services), epidemiology (number of episodes of depression and number of visits to doctors), marketing (number of sales of different products), sports (number of goals scored by opponent teams), econometrics (job switching voluntarily or involuntarily), etc. As mentioned, a typical example of such dependence arises in the traffic accidents where the extent of physical injuries may lead to fatalities. Leiter and Hamdan [1] suggested bivariate probability models applicable to traffic accidents and fatalities. A similar problem was addressed by Cacoullos and Papageorgiou [2]. Several other studies defined and studied the bivariate Poisson distribution [3–10].

The bivariate Poisson distributions have been developed following various assumptions. Among those, the most comprehensive one has been proposed by Kocherlakota and Kocherlakota [11]. The bivariate Poisson form is shown by using a trivariate reduction method [8] allowing for correlation between the variables, which is considered as a nuisance parameter. This bivariate Poisson regression is used by Jung and Winkelmann [8], Vernic [12] and Karlis and Ntzoufras [9, 13] among others. However, there is no attempt for modelling right truncated bivariate Poisson regression model so far which can play an important role for analyzing bivariate count data arising from a wider range of problems emerging in various fields.

Leiter and Hamdan [1] suggested joint distributions for number of accidents and number of fatalities by Poisson-Bernoulli (or Binomial) and Poisson-Poisson distribution. An alternative Poisson-Binomial model was proposed by Cacoullos and Papageorgiou [2]. In this paper, we have used the Poisson-Poisson distribution and the model for covariate dependence based on extended generalized linear model proposed by Islam and Chowdhury [14]. The marginal and conditional models are employed which are expressed in terms of the family of exponential distributions. Then a generalized linear model for this bivariate form is developed and the link functions are identified and expressed as functions of covariates. In some cases, we need to use right truncated Poisson distribution for both the outcome variables due to restrictions in the definition of variables. A model is shown for taking account of right truncated bivariate Poisson and a generalized bivariate count regression model is proposed using marginal-conditional approach. As both the tests for goodness of fit and over(under)dispersion are of prime concern in dealing with these models, some test procedures are shown in this paper. Gurmu and Trivedi [15, 16] suggested overdispersion tests for truncated Poisson regression models for univariate case. In this study, Gurmu and Trivedi [15] test is extended for bivariate Poisson for both untruncated and right truncated Poisson regression. For testing the goodness of fit tests for right truncated bivariate data, the test proposed by Islam and Chowdhury [14] is extended for truncated data. The estimation and test procedures are applied to health data, namely, the Health and Retirement Study [17] on number of health conditions ever had and utilization of healthcare services. Both the models with or without truncation are fitted to the same data and goodness of fit tests are applied to compare the models.

## The bivariate Poisson-Poisson model

Let *Y*_1_ be the number of occurrences of the first event in a given interval follow a Poisson distribution with parameter *λ*_1_ and the probability of the second event, *Y*_2_, for given *Y*_1_, where *Y*_2_ = *Y*_21_ + … + *Y*_2*y*_1__, be Poisson with parameter, *λ*_1_*y*_1_, *y*_21_, …, *y*_2*y*_1__ = 0, 1, …. Then the marginal, conditional and joint distributions of *Y*_1_ and *Y*_2_ can be shown as follows

$$\begin{array}{c} g\left(y_{1}\right)=\frac{e^{-\lambda_{1}}\lambda_{1}^{y_{1}}}{y_{1}!},y_{1}=0,1,... \end{array}$$

$$\begin{array}{c} g\left(y_{2}|y_{1}\right)=\frac{e^{-\lambda_{2}y_{1}}{\left(\lambda_{2}y_{1}\right)}^{y_{2}}}{y_{2}!},y_{2}=0,1,... \end{array}$$

Multiplying the marginal and conditional distributions shown above, we obtain the following joint distribution

$$\begin{array}{c} g\left(y_{1},y_{2}\right)=g\left(y_{2}|y_{1}\right)g\left(y_{1}\right)=\frac{e^{-\lambda_{1}}\lambda_{1}^{y_{1}}e^{-\lambda_{2}y_{1}}{\left(\lambda_{2}y_{1}\right)}^{y_{2}}}{\left(y_{1}!y_{2}!\right)},y_{1}=0,1,... \end{array}$$ (1)

The probability mass function for *Y*_2_ is

$$\begin{array}{c} g_{2}\left(y_{2}\right)=\frac{e^{-\lambda_{1}}\lambda_{2}^{y_{2}}}{y_{2}!}\sum_{y_{1}=0}^{\infty }\frac{{\left(\lambda_{1}e^{-\lambda_{2}}\right)}^{y_{1}}y_{1}^{y_{2}}}{y_{1}!},\;y_{2}=0,1,... \end{array}$$ (2)

It is noteworthy that in the proposed model, the marginal models for *Y*_1_ and *Y*_2_ do not allow same joint probability mass function from *g*(*y*_2_)*g*(*y*_1_ ∣ *y*_2_) and *g*(*y*_1_)*g*(*y*_2_ ∣ *y*_1_) which is expected as we observe from corresponding marginal probabilities.

The bivariate Poisson-Poisson expression in Eq (1) can be expressed as bivariate exponential form as follows

$$\begin{array}{c} g\left(y_{1},y_{2}\right)=e^{\left\{y_{1}\ln\lambda_{1}+y_{2}\ln\lambda_{2}-\lambda_{1}-\lambda_{2}y_{1}+y_{2}\ln y_{1}-\ln y_{1}!-\ln y_{2}!\right\}}. \end{array}$$ (3)

The link functions are ln *λ*_1_ = *x*′*β*_1_, ln *λ*_2_ = *x*′*β*_2_, where, $x^{\prime }=\left(1,x_{1},...,x_{p}\right),\beta_{1}^{\prime }=\left(\beta_{10},\beta_{11},...,\beta_{1p}\right),\beta_{2}^{\prime }=\left(\beta_{20},\beta_{21},...,\beta_{2p}\right).$

Hence, we can show that $\lambda_{1}=e^{x^{\prime }\beta_{1}}$, and $\lambda_{2}=e^{x^{\prime }\beta_{2}}.$ It is noteworthy that *E*(*Y*_1_) = *μ*_1_ = *λ*_1_ and *E*(*Y*_2_) = *μ*_2_ = *λ*_1_*λ*_2_. Hence, $\mu_{1}\left(\beta_{1}\right)=e^{x^{\prime }\beta_{1}}$ and $\mu_{2}\left(\beta_{1},\beta_{2}\right)=e^{x^{\prime }\beta_{1}+x^{\prime }\beta_{2}}.$

The log likelihood function is

$$\begin{array}{c} \ln L=\sum_{i=1}^{n}\left\{y_{1i}x_{i}^{\prime }\beta_{1}+y_{2i}x_{i}^{\prime }\beta_{2}-e^{x_{i}^{\prime }\beta_{1}}-e^{x_{i}^{\prime }\beta_{2}}y_{1i}+y_{2i}\ln y_{1i}-\ln y_{1i}!-\ln y_{2i}!\right\} \end{array}$$ (4)

## Bivariate right truncated Poisson-Poisson model

Let *Y*_1_ be the number of occurrences of the first event in a given interval which has a truncated Poisson distribution with mass function

$$\begin{array}{c} g_{1}\left(y_{1}\right)=c_{1}\frac{e^{-\lambda_{1}}\lambda_{1}^{y_{1}}}{y_{1}!},\;y_{1}=0,1,...,k_{1} \end{array}$$ (5)

where

$$\begin{array}{c} c_{1}=\frac{1}{\sum_{y_{1}=0}^{k_{1}}\frac{e^{-\lambda_{1}}\lambda_{1}^{y_{1}}}{y_{1}!}}. \end{array}$$

Then the probability of the second event, *Y*_2_, for given *y*_1_, where *Y*_2_ = *Y*_21_ + … + *Y*_2*y*_1__, the total number of second event cases among the truncated *y*_1_ events in the specified time interval, is:

$$\begin{array}{c} g\left(y_{2}|y_{1}\right)=c_{2}\frac{e^{-\lambda_{2}y_{1}}{\left(\lambda_{2}y_{1}\right)}^{y_{2}}}{y_{2}!},\;y_{2}=0,1,...,k_{y_{1}} \end{array}$$ (6)

where

$$\begin{array}{c} c_{2y_{1}}=\frac{1}{\sum_{y_{2}=0}^{k_{2y_{1}}}\frac{e^{-\lambda_{2}y_{1}}{\left(\lambda_{2}y_{1}\right)}^{y_{1}}}{y_{2}!}}. \end{array}$$

Multiplying the conditional and marginal probability functions, the joint distribution of *Y*_1_ and *Y*_2_ can be shown as follows

$$\begin{array}{c} g\left(y_{1},y_{2}\right)=g\left(y_{2}|y_{1}\right)g\left(y_{1}\right)=c_{1}c_{2}e^{-\lambda_{1}}\lambda_{1}^{y_{1}}e^{-\lambda_{2}y_{1}}{\left(\lambda_{2}y_{1}\right)}^{y_{2}}/\left(y_{1}!y_{2}!\right). \end{array}$$ (7)

The truncated Poisson-Poisson expression can be expressed as bivariate exponential form as follows

$$\begin{array}{c} g\left(y_{1},y_{2}\right)=e^{\left\{y_{1}\ln\lambda_{1}+y_{2}\ln\lambda_{2}-\lambda_{1}-\lambda_{2}y_{1}+y_{2}\ln y_{1}-\ln y_{1}!-\ln y_{2}!+\ln c_{1}+\ln c_{2}\right\}}. \end{array}$$ (8)

The expected value of *Y*_1_ can be shown as

$$\begin{array}{c} \mu_{Y_{1}}=E\left(Y_{1}\right)=\sum_{y_{1}=0}^{k1}\left[y_{1}\left(\frac{e^{-\lambda_{1}}\lambda_{1}^{y_{1}}}{y_{1}!}\right)\left(\frac{1}{\sum_{y_{1}=0}^{k_{1}}\frac{e^{-\lambda_{1}}\lambda_{1}^{y_{1}}}{y_{1}!}}\right)\right]=\frac{\lambda_{1}\Gamma \left(k1,\lambda_{1}\right)k1}{\Gamma (k1+1,\lambda_{1})}, \end{array}$$

where incomplete gamma function approximations are obtained based on relationships between cumulative Poisson function and incomplete gamma functions shown in Abramowitz and Stegun Chapters 6, 7 and 26 [18].

Similarly the conditional expected value for *Y*_2_ can be expressed as

$$\begin{array}{ccc} \mu_{Y_{2}/y_{1}} & = & E\left(Y_{2}|y_{1}\right)=\sum_{y_{2=0}}^{k2}\left[y_{2}\left(\frac{e^{-\lambda_{2}y_{1}}{\left(\lambda_{2}y_{1}\right)}^{y_{2}}}{y_{2}!}\right)\left(\frac{1}{\sum_{y_{2}=0}^{k_{2}}\frac{e^{-\lambda_{2}y_{1}}{\left(\lambda_{2}y_{1}\right)}^{y_{2}}}{y_{2}!}}\right)\right]\\ & = & \frac{\left(\lambda_{2}y_{1}\right)\Gamma \left(k2,\lambda_{2}y_{1}\right)k2}{\Gamma (k2+1,\lambda_{2}y_{1})}. \end{array}$$

The link functions in model Eq (7) are ln *λ*_1_ = *x*′*β*_1_, ln *λ*_2_ = *x*′*β*_2_, where $\beta^{\prime }=\left(\beta_{1}^{\prime },\beta_{2}^{\prime }\right)$, *x*′ = (1, *x*_1_, …, *x*_*p*_), ${\beta_{1}}^{\prime }=\left(\beta_{10},\beta_{11},...,\beta_{1p}\right)$, $\beta_{2}^{\prime }=\left(\beta_{20},\beta_{21},...,\beta_{2p}\right)$ and the log likelihood and is shown below:

$$\begin{array}{ccc} \ln L & = & \sum_{i=1}^{n}\{y_{1i}\ln\lambda_{1}+y_{2i}\ln\lambda_{2}-\lambda_{1}-\lambda_{2}y_{1i}+y_{2i}\ln y_{1i}-\ln y_{1i}!-\ln y_{2i}!\\ & & +lnc_{1}+\ln c_{2y_{1}}\\ & = & \sum_{i=1}^{n}\{y_{1i}x_{1i}^{\prime }\beta_{1}+y_{2i}x_{1i}^{\prime }\beta_{2}-e^{x_{1i}^{\prime }\beta_{1}}-e^{x_{1i}^{\prime }\beta_{2}}y_{1i}+y_{2i}\ln y_{1i}-\ln y_{1i}!-\ln y_{2i}!\\ & & +lnc_{1}+\ln c_{2y_{1}}\}. \end{array}$$ (9)

## Test for goodness of fit and under(Over) dispersion

In Sections 2 and 3, the proposed generalized models for untruncated or right truncated data are based on marginal and conditional probabilities as functions of covariates, we need to use a test for goodness of fit suitable for such models. A test for goodness of fit is proposed for the bivariate Poisson-Poisson model [14] using marginal and conditional means is:

$$\begin{array}{ccc} & & T_{1}=\sum_{y_{1}=0}^{k_{1}}T_{1}y_{1}=\\ & & \;\sum_{y_{1}=0}^{k_{1}}{\left(\begin{array}{ccc} {\bar{y}}_{1} & - & {\hat{\mu }}_{u,y_{1}}\\ {\bar{y}}_{2}|y_{1} & - & {\hat{\mu }}_{u,y_{2}|y_{1}} \end{array}\right)}^{\prime }{\left(\begin{array}{cc} \sum_{i=1}^{n_{y_{1}}}{\hat{\lambda }}_{1i}/n_{y_{1}} & 0\\ 0 & \sum_{i=1}^{n_{y_{1}}}{\hat{\lambda }}_{2i}y_{1}/n_{y_{1}} \end{array}\right)}^{-1}\left(\begin{array}{ccc} {\bar{y}}_{1} & - & {\hat{\mu }}_{u,y_{1}}\\ {\bar{y}}_{2}|y_{1} & - & {\hat{\mu }}_{u,y_{2}|y_{1}} \end{array}\right) \end{array}$$ (10)

where *T*_1_*y*_1_ can be shown asymptotically as a *χ*^2^ with 2 degrees of freedom. Hence *T*_1_ is distributed asymptotically as *χ*^2^ with 2(*k*_1_ + 1) degrees of freedom as each *T*_1_*y*_1_ is independent chi-square, *k*_1_ + 1 is the number of groups of distinct *y*_1_ values such as *Y*_1_ = 0 with frequency *n*_0_, *Y*_1_ = 1 with frequency *n*_1_, …, *Y*_1_ = *k*_1_ with frequency *n*_*k*_1__, ${\hat{\mu }}_{y_{1}}=\sum_{i=1}^{n_{y_{1}}}{\hat{\lambda }}_{1i}/n_{y_{1}}$, and ${\hat{\mu }}_{y_{2}|y_{1}}=\sum_{i=1}^{n_{y_{1}}}{\hat{\lambda }}_{2i}y_{1}/n_{y_{1}}$. Here ${\hat{\lambda }}_{1i}=e^{x_{i}^{\prime }{\hat{\beta }}_{1}}$, and ${\hat{\lambda }}_{2}=e^{x_{i}^{\prime }{\hat{\beta }}_{2}}$.

Similarly, the goodness of fit test statistic as shown in Eq (10) can be modified for the bivariate truncated model too. In this case, the null and alternative hypotheses are: *H*_0_: the data follow the proposed truncated bivariate Poisson model and, *H*_1_: the data do not follow the proposed truncated bivariate Poisson model. The modified test statistic for testing the goodness of fit is:

$$\begin{array}{c} \sum_{y_{1}=0}^{k_{1}}{\left(\begin{array}{ccc} {\bar{y}}_{1} & - & {\hat{\mu }}_{t,y_{1}}\\ {\bar{y}}_{2}|y_{1} & - & {\hat{\mu }}_{t,y_{2}|y_{1}} \end{array}\right)}^{\prime }{\left(\begin{array}{cc} \sum_{i=1}^{n_{y_{1}}}{\hat{V}}_{1i}/n_{y_{1}} & 0\\ 0 & \sum_{i=1}^{n_{y_{1}}}{\hat{V}}_{2i|1}/n_{y_{1}} \end{array}\right)}^{-1}\left(\begin{array}{ccc} {\bar{y}}_{1} & - & {\hat{\mu }}_{t,y_{1}}\\ {\bar{y}}_{2}|y_{1} & - & {\hat{\mu }}_{t,y_{2}|y_{1}} \end{array}\right) \end{array}$$ (11)

where *T*_1_*y*_1_ can be shown asymptotically as a *χ*^2^ with 2 degrees of freedom. Hence *T*_1_ is distributed asymptotically as *χ*^2^ with 2(*k*_1_ + 1) degrees of freedom as each *T*_1_*y*_1_ is independent chi-square, *k*_1_ + 1 is the number of groups of distinct *y*_1_ values such as *Y*_1_ = 0 with frequency *n*_0_, *Y*_1_ = 1 with frequency *n*_1_, …, *Y*_1_ = *k*_1_ with frequency *n*_*k*_1__, ${\hat{\mu }}_{y_{1}}=\frac{{\hat{\lambda }}_{1}\Gamma \left(k_{1},{\hat{\lambda }}_{1}\right)k_{1}}{\Gamma (k_{1}+1,{\hat{\lambda }}_{1})}$, ${\hat{\mu }}_{y_{2}|y_{1}}=\frac{\left({\hat{\lambda }}_{2}\;y_{1}\right)\Gamma \left(k_{2y_{1}},{\hat{\lambda }}_{2}\;y_{1}\right)k_{2y_{1}}}{\Gamma (k_{2y_{1}}+1,{\hat{\lambda }}_{2}\;y_{1})}$, *V*_1_ and *V*_2|1_ are the variances of *Y*_1_ and *Y*_2_ given *Y*_1_ respectively (see Appendix). Using ${\hat{\lambda }}_{1i}=e^{{x^{\prime }}_{i}{\hat{\beta }}_{1}^{*}},\;{\hat{\lambda }}_{2i}=e^{{x^{\prime }}_{i}{\hat{\beta }}_{2}^{*}}$, we obtain ${\hat{V}}_{1i}$ and ${\hat{V}}_{2i|1}$ from *V*_1_ and *V*_2|1_ for *i* = 1, 2, …, *n*_*y*_1__.

We can modify the test for goodness of fit as shown in Eqs (10) and (11) to develop test statistics for over(under)dispersion. As overdispersion and underdispersion may influence the fit of the proposed untruncated Poisson regression models, we have used the method of moments estimator [14, 19–21] to estimate dispersion parameter for untruncated model, *φ*_*u*,*r*_ where ${\hat{\varphi }}_{u,r}=\frac{1}{n-p}\sum_{i=1}^{n}\left[\frac{{\left(y_{ri}-{\hat{\mu }}_{u,ri}\right)}^{2}}{V({\hat{\mu }}_{u,ri})}\right]=\frac{\chi_{r,n-p}^{2}}{n-p},\;r=1,2,$ where $V\left({\hat{\mu }}_{u,ri}\right)={\hat{\mu }}_{u,ri}.$

Using the mean, variance and correction factor [15] for right truncated Poisson marginal and conditional models, we can define ${\hat{\mu }}_{t,ri}={\hat{\lambda }}_{ri}+{\hat{\delta }}_{ri}$ and $\hat{V}\left({\hat{\mu }}_{t,ri}\right)={\hat{\lambda }}_{ri}-{\hat{\delta }}_{ri}\left({\hat{\mu }}_{ri}-k_{r}-1\right)$ where ${\hat{\delta }}_{ri}={\hat{\mu }}_{ri}-{\hat{\lambda }}_{ri}=-{\hat{\lambda }}_{ri}.\hat{\alpha }\left(k_{r},{\hat{\lambda }}_{ri}\right)$, $\hat{\alpha }\left(k_{r},\lambda_{ri}\right)=\frac{h(k_{r},\lambda_{ri})}{1-H(k_{r},\lambda_{ri})}$, *h*(*k*_*r*_, *λ*_*ri*_) = *P*(*Yri* = *k*_*r*_), *H*(*k*_*r*_, *λ*_*ri*_) = *P*(*Yri* ≤ *k*_*r*_) and then using these values we can estimate *φ*_*r*_ for the truncated model.

A test for over(under)dispersion, T2, is proposed assuming adjusted variances for the marginal mean of Y1 and conditional means of Y2 given Y1 = y1. If there is over(under)dispersion, then the test results in acceptance of the null hypothesis that the expected values are equal to the adjusted variances.

Then *T*_2_ for untruncated bivariate Poisson regression model is:

$$\begin{array}{ccc} & & T_{2}=\sum_{y_{1}=0}^{k_{1}}T_{2}y_{1}=\\ & & \;{\left(\begin{array}{c} {\bar{y}}_{1}-\hat{\mu }{}_{u,y_{1}}^{*}\\ {\bar{y}}_{2}|y_{1}-{\hat{\mu }}_{u,y_{2}|y_{1}}^{*} \end{array}\right)}^{\prime }{\left(\begin{array}{cc} \frac{{\hat{\phi }}_{u,1}\sum_{i=1}^{n_{y_{1}}}{\hat{\lambda }}_{1i}}{n_{y_{1}}} & 0\\ 0 & \frac{{\hat{\phi }}_{u,2}\sum_{i=1}^{n_{y_{1}}}{\hat{\lambda }}_{2i}y_{1}}{n_{y_{1}}} \end{array}\right)}^{-1}\left(\begin{array}{c} {\bar{y}}_{1}-\hat{\mu }{}_{u,y_{1}}^{*}\\ {\bar{y}}_{2}|y_{1}-{\hat{\mu }}_{u,y_{2}|y_{1}}^{*} \end{array}\right) \end{array}$$ (12)

where *T*_2_*y*_1_ can be shown asymptotically as a *χ*^2^ with 2 degrees of freedom. Hence *T*_2_ is distributed asymptotically as *χ*^2^ with 2(*k*_1_ + 1) degrees of freedom as each *T*_2_*y*_1_ is independent chi-square, ${\hat{\mu }}_{u,y_{1}}^{*}={\hat{\phi }}_{u,1}{\hat{\mu }}_{u,y_{1}}$, and ${\hat{\mu }}_{u,y_{2}|y_{1}}^{*}={\hat{\phi }}_{u,2}{\hat{\mu }}_{u,y_{2}|y_{1}},$ *u* denotes untruncated model, ${\hat{\lambda }}_{1i}=e^{x_{i}^{\prime }{\hat{\beta }}_{1}}$, ${\hat{\lambda }}_{2}=e^{x_{i}^{\prime }{\hat{\beta }}_{2}}$ and *k*_1_ + 1 is the number of distinct counts observed for *Y*_1_. Similarly, for right truncated model *T*_2_ can be defined as follows:

$$\begin{array}{ccc} & & T_{2}=\sum_{y_{1}=0}^{k_{1}}T_{2}y_{1}=\\ & & \;\sum_{y_{1}=0}^{k_{1}}{\left(\begin{array}{c} {\bar{y}}_{1}-\hat{\mu }{}_{t,y_{1}}^{*}\\ {\bar{y}}_{2}|y_{1}-{\hat{\mu }}_{t,y_{2}|y_{1}}^{*} \end{array}\right)}^{\prime }{\left(\begin{array}{cc} \frac{{\hat{\phi }}_{t,1}\sum_{i=1}^{n_{y_{1}}}{\hat{V}}_{1i}}{n_{y_{1}}} & 0\\ 0 & \frac{{\hat{\phi }}_{t,2}\sum_{i=1}^{n_{y_{1}}}V_{2i|1}}{n_{y_{1}}} \end{array}\right)}^{-1}\left(\begin{array}{c} {\bar{y}}_{1}-\hat{\mu }{}_{t,y_{1}}^{*}\\ {\bar{y}}_{2}|y_{1}-{\hat{\mu }}_{t,y_{2}|y_{1}}^{*} \end{array}\right) \end{array}$$ (13)

where *T*_2_*y*_1_ can be shown asymptotically as a *χ*^2^ with 2 degrees of freedom. Hence *T*_2_ is distributed asymptotically as *χ*^2^ with 2(*k*_1_ + 1) degrees of freedom as each *T*_2_*y*_1_ is independent chi-square, ${\hat{\mu }}_{t,y_{1}}^{*}={\hat{\varphi }}_{t,1}{\hat{\mu }}_{t,y_{1}}^{,}\;and\;\;{\hat{\mu }}_{t,y_{2}|y_{1}}^{*}={\hat{\varphi }}_{t,2}{\hat{\mu }}_{t,y_{2}|y_{1}}$, ${\hat{\lambda }}_{1i}=e^{x_{i}^{\prime }{\hat{\beta }}_{1}^{*}}$ and ${\hat{\lambda }}_{2i}=e^{x_{i}^{\prime }{\hat{\beta }}_{2}^{*}}$, *t* denotes truncated model.

In the above tests for the goodness of fit, specific models (proposed models) are tested. Hence, the test for goodness of fit may be termed equivalently as the test for specification of the proposed models.

## Test for model selection

Voung [22] developed a general test for nonnested models. This statistic tests the hypothesis that two nonnested parametric models are equally distant in the Kullback-Leibler sense from the true data distribution. This test statistic is used to select a parsimonuous model. Consider following exponential density form for generalized linear models:

$$\begin{array}{c} f\left(y,\theta \right)=e^{\left[\left\{y\theta -b(\theta )\}/a(\phi )+c(y,\phi \right)\right]}, \end{array}$$

where *θ* is the natural link function which is a function of expected value of *Y*, *b*(*θ*) is a function of *θ*, *a*(*ϕ*) is dispersion parameter and *c*(*y*, *ϕ*) is a function of *y* and *ϕ*. We can obtain expected value and variance of *Y* from *E*(*Y*) = *b*′(*θ*) and *Var*(*Y*) = *a*(*ϕ*)*b*′′(*θ*) where *b*′′(*θ*) is the variance function, *Var*[*E*(*Y*)]. From the probability function we can show that *θ* = ln *μ*_1_, *E*(*Y*_1_) = *μ*_1_, and *Var*(*Y*_1_) = *μ*_1_/*ϕ*_1_. Similarly, using the conditional probability function for *Y*_2_ given *Y*_1_ = *y*_1_, we find *θ* = ln *μ*_2_*y*_1_, *E*(*Y*_2_|*y*_1_) = *μ*_2_*y*_1_ and *Var*(*Y*_2_|*y*_1_) = *μ*_2_*y*_1_/*ϕ*_2_.

The Voung test is a *t* or standard normal test for large sample where

$$\begin{array}{c} V=\frac{\bar{m}\sqrt{n}}{s_{m}},\;\bar{m}=\frac{\sum_{i=1}^{n}m_{i}}{n},\;m_{i}=\ln\left[f\left(y_{1i},y_{2i};\theta \right)/g\left(y_{1i},y_{2i};\theta^{\prime }\right)\right], \end{array}$$

where *f*(*y*_1*i*_, *y*_2*i*_;*θ*) is the probability function for model with parameter vector *θ* and *g*(*y*_1*i*_, *y*_2*i*_;*θ*′) the probability function for model with parameter vector *θ*′ and

$$\begin{array}{c} s_{m}^{2}=\frac{1}{n}\left[\sum_{i=1}^{n}{\left\{\ln\left(\frac{f(y_{1i},y_{2i};\theta )}{g(y_{1i},y_{2i};\theta^{\prime })}\right)\right\}}^{2}-\frac{1}{n}{\left[\sum_{i=1}^{n}\left\{\ln\left(\frac{f(y_{1i},y_{2i};\theta )}{g(y_{1i},y_{2i};\theta^{\prime })}\right)\right\}\right]}^{2}\right] \end{array}$$

Adjusted Vuong test is

$$\begin{array}{c} V=\frac{{\bar{m}}^{\prime }\sqrt{n}}{s_{m^{\prime }}},\;{\bar{m}}^{\prime }=\frac{\sum_{i=1}^{n}m_{i}^{\prime }}{n},\;\ln\left[\frac{f(y_{1i},y_{2i};\theta )}{g(y_{1i},y_{2i};\theta^{\prime })}\right]-\frac{(p-q)-\ln n}{2n}, \end{array}$$

where *p* = number of parameters in numerator in model *f*(*y*_1*i*_, *y*_2*i*_;*θ*) and *q* = number of parameters in denominator in model *g*(*y*_1*i*_, *y*_2*i*_;*θ*′). If *V* > 1.96 then model in the numerator is favored and if *V* < −1.96 then model in the denominator is favored.

## Application

The models proposed in this paper are applied to the tenth wave of the Health and Retirement Study [17]. The outcome variables are number of conditions ever had (*Y*_1_) as mentioned by doctors and utilization of healthcare services (*Y*_2_) where utilization of healthcare services include services from hospital, nursing home, doctor, and home care. The explanatory variables are: gender (1 male, 0 female), age (in years), race (1 Hispanic, 0 others) and veteran status (1 yes, 0 no). The sample size is 5568.

Table 1 displays the average number of conditions (*Y*_1_) and utilization of health care services (*Y*_2_) and sample variances. The average number of conditions is 2.63 and the variance (2.05) appears to be lower than the mean. The average number of utilization of health care services is 0.77 and the corresponding variance is slightly higher (0.79). The measure of correlation between number of conditions and utilization of health care services can be estimated by $r={\left[{\hat{\lambda }}_{2}/\left({\hat{\lambda }}_{2}+1\right)\right]}^{\frac{1}{2}}$ where ${\hat{\lambda }}_{2}=\frac{{\bar{y}}_{2}}{{\bar{y}}_{1}}$ (see Leiter and Hamdan, [1]). The estimated correlation is 0.48 and the corresponding standard error is very small indicating significant association between the bivariate counts. The estimated standard error of r is $\hat{se(r)}={\left[{\bar{y}}_{1}^{2}/{\left\{4n\left({\bar{y}}_{1}+{\bar{y}}_{2}\right)\right\}}^{3}\right]}^{\frac{1}{2}}.$

**Table 1:** Descriptive measures of outcome variables.

Measures	Health and Retirement Study (HRS, 2010)
${\bar{y}}_{1}$	2.642716
$\hat{Var}\left(Y_{1}\right)$	2.117924
${\bar{y}}_{2}$	0.769176
$\hat{Var}\left(Y_{2}\right)$	0.792021

The dispersion parameters for number of conditions (*Y*_1_) and utilization of health care services (*Y*_2_) are 0.80 and 1.05, respectively. A test for over(under)dispersion using *T*_2_ indicates that there might be statistically significant over(under) dispersion in the bivariate count data. This indicates slight overdispersion for utilization of health care services and underdispersion for number of conditions. Using the mean, variance and correction factor [15] for right truncated Poisson marginal and conditional models, we can estimate the dispersion parameters.

Table 2 displays estimates of the parameters of the marginal and conditional models for the untruncated model as well as tests for significance of the parameters with and without considering adjustment for over or under dispersion. The selected explanatory variables in the models are gender, age, race and veteran status. All the variables except race show statistically significant association with number of conditions. From the marginal model for number of conditions it appears that the number of conditions is significantly lower for males compared to females but age and veteran status are positively associated with number of conditions. This shows that the number of conditions in health increases steadily with age, as expected, and the veterans have higher number of conditions compared to non-veterans. There is no significant difference in number of conditions by race. As there is evidence of under dispersion in the number of conditions, an adjustment is made using the dispersion parameter, After adjustment, the standard errors are smaller which are displayed in Table 2 and p-values appear to be smaller. We observe that the conditional model for utilization of healthcare services for given number of conditions indicate significant positive association with gender (male = 1, female = 0) and veteran status (yes = 1, no = 0) while age and race (race = 1 for Hispanic, race = 0, otherwise) show negative association. Although number of conditions is lower for males, utilization of healthcare services appears to be higher among males compared to females. Another important finding reveals that the utilization of healthcare services reduces significantly with age for given number of indications which implies that the with ncreased age elderly people do not take healthcare services from different sources. Race is not associated significantly with number of conditions but utilization of healthcare services show a negative association with race indicating lower utilization for Hispanics as compared to other races. There is positive association between utilization of healthcare services and veteran status, similar to the association found with number of conditions observed from the marginal model.

**Table 2:** Parameter estimates for the bivariate poisson models using the data on number of conditions and utilization of healthcare services (HRS, 2010).

Variables	Estimate	S.E.	z-value	p-value	$\sqrt{\varphi_{r}V\left(\hat{\beta }\right)}$	p-value
Marginal model for (*Y*_1_)						
*β*_10_	−0.055	0.1953	−0.281	0.779	0.1720	0.750
Gender	−0.055	0.0214	−2.573	0.010	0.0189	0.003
Age (years)	0.014	0.0026	5.315	0.000	0.0023	0.000
Race	0.007	0.0288	0.234	0.815	0.0254	0.790
Veteran status	0.048	0.025	1.937	0.053	0.0220	0.028
Conditional model for (*Y*_2_)						
*β*_20_	0.265	0.3627	0.730	0.466	0.3713	0.476
Gender	0.345	0.0385	8.953	0.000	0.0395	0.000
Age (years)	−0.023	0.0049	−4.608	0.000	0.0050	0.000
Race	−0.174	0.0582	−2.997	0.003	0.0590	0.003
Veteran status	0.093	0.0423	2.208	0.027	0.0432	0.031
Gender (male = 1, female = 0); Race (Hispanic = 1, others = 0); Veteran status (yes = 1, no = 0).						

The estimates for the right truncated bivariate Poisson model are displayed in Table 3. The results are generally similar to the fit of the untruncated model displayed in Table 2 but estimates of the parameters for the right truncated model show substantial difference from that of the untruncated model and it is more evident for the conditional part of the joint model. Let us denote *β*_1_∗ = (*β*_11_, …, *β*_1*p*_)′, *β*_2_∗ = (*β*_21_, …, *β*_2*p*_)′, $\beta *=({\beta_{1}}^{\prime },{\beta_{2}}^{\prime })$ then for the overall test for the null hypothesis *H*_0_: *β*∗ = 0, the following likelihood ratio test is used $\chi^{2}=-2\left[\ln L\left({\hat{\beta }}_{10},{\hat{\beta }}_{20}\right)-\ln L\left(\hat{\beta }\right)\right]$ where $\beta =({\beta_{1}}^{\prime },{\beta_{2}}^{\prime })$. Here the likelihood ratio test statistic is asymptotically chi square with 2p degrees of freedom. Both the models for untruncated and truncated appear statistically significant. The estimated regression coefficients for gender for the untruncated model is 0.345 compared to 0.453 in the truncated model. Similarly, the estimated regression coefficient for veteran status is 0.093 and 0.219 in the untruncated and truncated models respectively. However, standard errors have not shown much change. The likelihood ratio test statistics show p-values less than 0.001 for both the untruncated and truncated models. It may be noted here that both the untruncated and truncated models are fitted to examine the changes in the estimation of parameters and tests attributable to truncation. Test for over (under) dispersion for both the models show that the fitted models are not significantly over or under dispersed.

**Table 3:** Parameter estimates for the truncated bivariate poisson models using the data on number of conditions and utilization of healthcare services (HRS, 2010).

Variables	Estimate	S.E.	z-value	p-value	$\sqrt{\varphi_{r}V\left(\hat{\beta }\right)}$	p-value
Marginal model for (*Y*_1_)						
*β*_10_	−0.180	0.2107	−0.854	0.393	0.1657	0.278
Gender	−0.063	0.0229	−2.765	0.006	0.0182	0.001
Age (years)	0.016	0.0029	5.719	0.000	0.0022	0.000
Race	0.008	0.0310	0.250	0.802	0.0244	0.751
Veteran status	0.056	0.0268	2.072	0.038	0.0213	0.009
Conditional model for (*Y*_2_)						
*β*_10_	0.888	0.3592	2.472	0.013	0.3126	0.005
Gender	0.453	0.0381	11.883	0.000	0.0337	0.000
Age (years)	−0.030	0.0049	−6.144	0.000	0.0043	0.000
Race	−0.249	0.0577	−4.310	0.000	0.0517	0.000
Veteran status	0.219	0.0418	5.252	0.000	0.0354	0.000
Gender (male = 1, female = 0); Race (Hispanic = 1, others = 0); Veteran status (yes = 1, no = 0).						

The estimated dispersion parameters for untruncated models, ${\hat{\varphi }}_{u,1}$ and ${\hat{\varphi }}_{u,2}$, are 0.80 and 1.05 respectively and from the truncated models ${\hat{\varphi }}_{t,1}$ and ${\hat{\varphi }}_{t,2}$ are 0.90 and 0.83 respectively (see Table 4). Adjusting for both untruncted models and truncated models, we observe that the test results for significance of the parameters remain similar.

**Table 4:** Model statistics for untruncated and truncated bivariate poisson models for Health and Retirement Study data (HRS) Data.

Statistics	Reduced Model		Full Model	
	Untruncated	Right truncated	Untruncated	Right truncated
Log Likelihood	−16707.66	−16596.21	−16586.07	–16500.71
AIC	33419.33	33196.42	33192.13	33021.41
BIC	33432.58	33209.67	33258.38	33087.66
T_1_ (D.F., p-value)	9.34 (12, 0.622)	11.89 (12, 0.455)	9.73 (12, 0.640)	11.80 (12, 0.462)
T_2_ (D.F., p-value)	12.26 (12, 0.425)	13.49 (12, 0.335)	12.03 (12, 0.444)	13.43 (12, 0.339)
LRT: Reduced vs. full model (D.F., p-value)	−	−	243.18 (8, 0.000)	191.01 (8, 0.000)
*φ*_1_	0.78	0.90	0.80	0.90
*φ*_2_	1.03	0.83	1.05	0.83
*V*: Voung test (p-value)		12.67 (0.000)		6.87 (0.000)

Table 4 summarizes the results for both untruncated and truncated bivariate Poisson regression models. We observe from goodness of fit test statistic, *T*_1_, that both untruncated and truncated bivariate Poisson regression models fit the data very well. In other words, the null hypotheses may be accepted in favor of the proposed models. To make a more precise decision about the choice of the better model, the Voung test is employed. We have used the Voung test for comparing non-nested models because non-nested models can not be compared using the classical likelihood ratio based tests. Table 4 shows comparison between untruncated and right truncated models and it is found that the bivariate right truncated Poisson model appears to be significantly better than the untruncated model (p<0.001).

## Conclusion

The use of bivariate count regression models can provide very useful insights for analyzing repeated measures data emerging from various fields including health. In this paper, a comprehensive methodology has been displayed to deal with different types of problems associated with bivariate count data. In this paper, a new model is proposed using the Poisson-Poisson marginal-conditional approach for both untruncated and truncated data. In reality, there may be influence of right truncation for analyzing such data and the application displayed in this paper clearly demonstrates substantial change in the estimates of regression parameters. A generalized linear model for the bivariate Poisson-Poisson regression model is developed for the right truncated data. The proposed model is applied to the Health and Retirement Study data on number of conditions and utilization of healthcare services. The problem of under or overdispersion in the count data is also examined and appropriate procedures for adjusting the standard errors are also shown. Tests for both goodness of fit and overdispersion have been used and it is observed that the models fit well to the bivariate count data and the extent of under or overdispersion are not found to be significant in the application to the data on number of conditions and utilization of healthcare services from the Health and Retirement Study. In order to select a better model, an extended test for non-nested models for bivariate count data is shown in this paper and it is found that the right truncated bivariate Poisson model is a better choice for the health data used in this study.

## Appendix

$$\begin{array}{c} E\left(Y_{1}\right)=\sum_{y_{1=0}}^{k_{1}}\left[y_{1}\left(\frac{e^{-\lambda_{1}}\lambda_{1}^{y_{1}}}{y_{1}!}\right)\left(\frac{1}{\sum_{y_{1}=0}^{k_{1}}\frac{e^{-\lambda_{1}}\lambda_{1}^{y_{1}}}{y_{1}!}}\right)\right]=\frac{\lambda_{1}\Gamma \left(k_{1},\lambda_{1}\right)k_{1}}{\Gamma (k_{1}+1,\lambda_{1})}. \end{array}$$

$$\begin{array}{ccc} E(Y_{1}^{2}) & = & \sum_{y_{1=0}}^{k1}\left[y_{1}^{2}\left(\frac{e^{-\lambda_{1}}\lambda_{1}^{y_{1}}}{y_{1}!}\right)\left(\frac{1}{\sum_{y_{1}=0}^{k_{1}}\frac{e^{-\lambda_{1}}\lambda_{1}^{y_{1}}}{y_{1}!}}\right)\right]=\frac{\Gamma (k1+1)}{\Gamma (k1+1,\lambda_{1})}(\lambda_{1}\left(\lambda_{1}+1\right)\\ & & -\frac{{\left(k1+1\right)}^{2}\lambda_{1}^{k1+1}hypergeometric\left(\left[1,k1+2\right],\left[k1+1,k1+1\right],\lambda_{1}\right)}{\left(k1+1\right)!e_{1}^{\lambda }}). \end{array}$$

$$\begin{array}{ccc} Var(Y_{1}) & = & V_{1}=E\left(Y_{1}^{2}\right)-{\left[E\left(Y_{1}\right)\right]}^{2}=-\frac{{\left(\lambda_{1}^{k1+1}\right)}^{2}{\left(\Gamma \left(k1,\lambda_{1}\right)\right)}^{2}k1^{2}}{{\left(\Gamma (k1+1,\lambda_{1})\right)}^{2}{\left(\lambda_{1}^{k1}\right)}^{2}}+\frac{\Gamma (k1+1)}{\Gamma (k1+1,\lambda_{1})}\\ & & (\lambda_{1}\left(\lambda_{1}+1\right)-\frac{{\left(k1+1\right)}^{2}\lambda_{1}^{k1+1}hypergeometric\left(\left[1,k1+2\right],\left[k1+1,k1+1\right],\lambda_{1}\right)}{\left(k1+1\right)!e^{\lambda_{1}}}). \end{array}$$

Similarly,

$$\begin{array}{c} E\left(Y_{2}\right)=\sum_{y_{2=0}}^{k2}\left[y_{2}\left(\frac{e^{-\lambda_{2}}\lambda_{2}^{y_{2}}}{y_{2}!}\right)\left(\frac{1}{\sum_{y_{2}=0}^{k_{2}}\frac{e^{-\lambda_{2}}\lambda_{2}^{y_{2}}}{y_{2}!}}\right)\right]=\frac{\lambda_{2}\Gamma \left(k2,\lambda_{2}\right)k2}{\Gamma (k2+1,\lambda_{2})}. \end{array}$$

$$\begin{array}{ccc} Var(Y_{2}) & = & E\left(Y_{2}^{2}\right)-{\left[E\left(Y_{2}\right)\right]}^{2}=-\frac{{\left(\lambda_{2}^{k2+1}\right)}^{2}{\left(\Gamma \left(k2,\lambda_{2}\right)\right)}^{2}k2^{2}}{{\left(\Gamma \left(k2+1,\lambda_{2}\right)\right)}^{2}{\left(\lambda_{2}^{k2}\right)}^{2}}+\frac{\Gamma (k2+1)}{\Gamma (k2+1,\lambda_{2})}\\ & & (\lambda_{2}\left(\lambda_{2}+1\right)-\frac{{\left(k2+1\right)}^{2}\lambda_{2}^{k1+1}hypergeometric\left(\left[1,k2+2\right],\left[k2+1,k2+1\right],\lambda_{2}\right)}{\left(k2+1\right)!e^{\lambda_{2}}}). \end{array}$$

$$\begin{array}{ccc} E(Y_{2}|y_{1}) & = & \sum_{y_{2}=0}^{k2}\left[y_{2}\left(\frac{e^{-\lambda_{2}y_{1}}{\left(\lambda_{2}y_{1}\right)}^{y_{2}}}{y_{2}!}\right)\left(\frac{1}{\sum_{y_{2}=0}^{k2}\frac{e^{-\lambda_{2}y_{1}}{\left(\lambda_{2}y_{1}\right)}^{y_{2}}}{y_{2}!}}\right)\right]\\ & = & \frac{\left(\lambda_{2}y_{1}\right)\Gamma \left(k2,\lambda_{2}y_{1}\right)k2}{\Gamma (k2+1,\lambda_{2}y_{1})}. \end{array}$$

$$\begin{array}{ccc} E(Y_{2}^{2}|y_{1}) & = & \sum_{y_{2}=0}^{k2}\left[y_{2}^{2}\left(\frac{e^{-\lambda_{2}y_{1}}{\left(\lambda_{2}y_{1}\right)}^{y_{2}}}{y_{2}!}\right)\left(\frac{1}{\sum_{y_{2}=0}^{k2}\frac{e^{-\lambda_{2}y_{1}}{\left(\lambda_{2}y_{1}\right)}^{y_{2}}}{y_{2}!}}\right)\right]=\frac{\Gamma (k2+1)}{\Gamma (k2+1,\lambda_{2}y_{1})}\\ & & (\lambda_{2}^{2}y_{1}^{2}+\lambda_{2}y_{1}-\frac{{\left(k2+1\right)}^{2}{\left(\lambda_{2}y_{1}\right)}^{k2+1}hypergeometric\left(\left[1,k2+2\right],\left[k2+1,k2+1\right],\lambda_{2}y_{1}\right)}{e^{\lambda_{2}y_{1}}\left(k2+1\right)!}). \end{array}$$

$$\begin{array}{ccc} & & Var\left(Y_{2}|y_{1}\right)=V_{2|1}=E\left(Y_{2}^{2}|y_{1}\right)-{\left[E\left(Y_{2}|y_{1}\right)\right]}^{2}=\\ & & \;-\frac{{\left({\left(\lambda_{2}y_{1}\right)}^{k2+1}\right)}^{2}{\left(\Gamma \left(k2,\lambda_{2}y_{1}\right)\right)}^{2}k2^{2}}{{\left(\Gamma \left(k2+1,\lambda_{2}y_{1}\right)\right)}^{2}{\left({\left(\lambda_{2}y_{1}\right)}^{k2}\right)}^{2}}+\frac{\Gamma (k2+1)}{\Gamma (k2+1,\lambda_{2}y_{1})}\\ & & \;(\lambda_{2}^{2}y_{1}^{2}+\lambda_{2}y_{1}-\frac{{\left(k2+1\right)}^{2}{\left(\lambda_{2}y_{1}\right)}^{k2+1}hypergeometric\left(\left[1,k2+2\right],\left[k2+1,k2+1\right],\lambda_{2}y_{1}\right)}{e^{\lambda_{2}y_{1}}\left(k2+1\right)!}). \end{array}$$
